# Integrative genetic and multi-omics analysis reveals the interleukin-6 receptor’s role in recurrent spontaneous abortion

**DOI:** 10.3389/fimmu.2025.1659251

**Published:** 2025-10-02

**Authors:** Weixu Ma, Zhongjia Gu, Qiwang Lin, Mingzhu Cao, Junmin Zhong, Xin Li, Hongyan Li, Yue Lin, Han Lin, Mengchang Xu, Jianqiao Liu, Lili Du, Ge Song

**Affiliations:** ^1^ Assisted Reproductive Technology Center, The Affiliated Foshan Women and Children Hospital, Guangdong Medical University, Foshan, China; ^2^ Hunan Provincial Key Laboratory of the Research and Development of Novel Pharmaceutical Preparations, Changsha Medical University, Changsha, China; ^3^ Department of Obstetrics and Gynecology, Guangdong Provincial Key Laboratory of Major Obstetric Diseases; Guangdong Provincial Clinical Research Center for Obstetrics and Gynecology; Guangdong-Hong Kong-Macao Greater Bay Area Higher Education Joint Laboratory of Maternal-Fetal Medicine; The Third Affiliated Hospital, Guangzhou Medical University, Guangzhou, China; ^4^ Department of Obstetrics and Gynecology, The First Affiliated Hospital of Guangzhou Medical University, Guangzhou, Guangdong, China; ^5^ Department of Obstetrics and Gynecology, Center for Reproductive Medicine; Guangdong Provincial Key Laboratory of Major Obstetric Diseases; Guangdong Provincial Clinical Research Center for Obstetrics and Gynecology; Guangdong-Hong Kong-Macao Greater Bay Area Higher Education Joint Laboratory of Maternal-Fetal Medicine; The Third Affiliated Hospital, Guangzhou Medical University, Guangzhou, China; ^6^ Department of Obstetrics, First Affiliated Hospital of Jinan University, Guangzhou, China; ^7^ Department of Obstetrics and Gynecology, Guangzhou Women and Children’s Medical Center, Guangzhou Medical, Guangzhou, China; ^8^ Department of Obstetrics and Gynecology, Guangdong Provincial Key Laboratory of Major Obstetric Diseases, The Third Affiliated Hospital of Guangzhou Medical University, Guangzhou, China

**Keywords:** Mendelian randomization, recurrent spontaneous abortion, plasma proteins, interleukin 6 receptor, phenome-wide association study

## Abstract

**Background:**

Recurrent spontaneous abortion (RSA) significantly impacts women’s health, yet the underlying biological mechanisms remain poorly defined. Understanding the molecular contributors to RSA is crucial for developing targeted interventions.

**Objective:**

This study aims to investigate the causal relationships between plasma proteins and RSA, focusing on the identification of potential therapeutic targets through multi-omic approaches.

**Methods:**

We utilized two-sample Mendelian randomization (MR) analyses integrating genome-wide association study (GWAS) data for both plasma proteins and RSA. Proteomic data were sourced from the UK Biobank-Plasma Proteome Project and deCODE Health Study. We further validated our findings through both bulk and single-cell RNA sequencing of clinical specimens, alongside quantitative real-time polymerase chain reaction and immunohistochemistry. A phenome-wide association study was also conducted to assess the safety and broader implications of identified targets.

**Results:**

Our analyses identified the interleukin 6 receptor (IL6R) as a key candidate, with elevated plasma levels correlating with increased RSA risk. Furthermore, IL6R was found to be upregulated in RSA-related endometrial and decidual tissues. The phenome-wide association study provided insights into potential side effects and additional therapeutic indications for IL6R.

**Conclusion:**

IL6R upregulation is mechanistically implicated in the pathogenesis of RSA, establishing it as a validated causal biomarker and a potentially actionable therapeutic target. This study not only highlights the role of IL6R in RSA but also supports its development into a therapeutic strategy with a comprehensive safety profile.

## Introduction

Recurrent spontaneous abortion (RSA), characterized by two or more consecutive miscarriages before 24 weeks of gestation with the same sexual partner, represents a significant challenge to women’s reproductive health (1, 2). Its incidence is estimated at 1% to 5% in the general population (1). RSA has a multifactorial etiology, including chromosomal abnormalities, uterine anatomical defects, autoimmune conditions, and endometrial dysfunction (3). Despite extensive investigation, the underlying biological mechanisms remain poorly understood, limiting effective clinical interventions. RSA not only increases the risk of adverse outcomes in subsequent pregnancies but also elevates the likelihood of long-term metabolic and cardiovascular complications, thereby substantially affecting women’s physical and psychological well-being (4–7).

Recently, plasma proteins are vital in physiological and pathological processes and are increasingly investigated in the context of diseases such as diabetes (8) and ankylosing spondylitis (9). Abnormal plasma protein levels, such as elevated antiphospholipid antibodies, are associated with a higher risk of RSA (10). Proteins, as downstream products of metabolic activities and modifiable entities through dietary or pharmacological means, act as vital biomarkers and prospective therapeutic targets for various disorders (11). Employing proteins intermediate phenotypes might provide crucial understanding of the mechanisms connecting plasma proteins with RSA. Nonetheless, results from observational research are frequently obscured by environmental influences, which constrain the ability to make strong causal determinations. Mendelian randomization (MR) offers a methodological advantage by using genetic variants as instrumental variables (IVs) to infer causality. By leveraging the random allocation of alleles during meiosis, akin to randomization in clinical trials, MR minimizes confounding and reverse causation, offering more robust evidence of causal relationships.

Increasing evidence underscores the importance of endometrial microenvironment remodeling during days 19–21 of the menstrual cycle, corresponding to the implantation window (LH + 7 to +9), in supporting successful implantation and pregnancy (12). This phase is marked by a highly receptive state, conducive to embryo implantation, both biochemically and structurally (13). Transcriptomic analyses during this window reveal dynamic shifts in immune tolerance, vascular remodeling, and steroid hormone responsiveness, with aberrations in these processes strongly implicated in RSA pathogenesis (1, 13–16). However, it remains unclear whether these microenvironmental changes are causally driven by systemic biomarker alterations or reflect localized endometrial dysfunction. To address this gap, we propose an integrative causal inference framework that combines population-level protein quantitative trait locus (pQTL) data with transcriptomic profiling of the endometrium during the implantation window. This approach employs MR to differentiate between systemic and tissue-specific causal pathways, while simultaneously identifying druggable targets supported by both genetic and functional evidence.

Ultimately, this study aims to advance our understanding of RSA pathogenesis and help in formulating targeted clinical strategies.

## Materials and methods

### Study design

**Figure 1:**
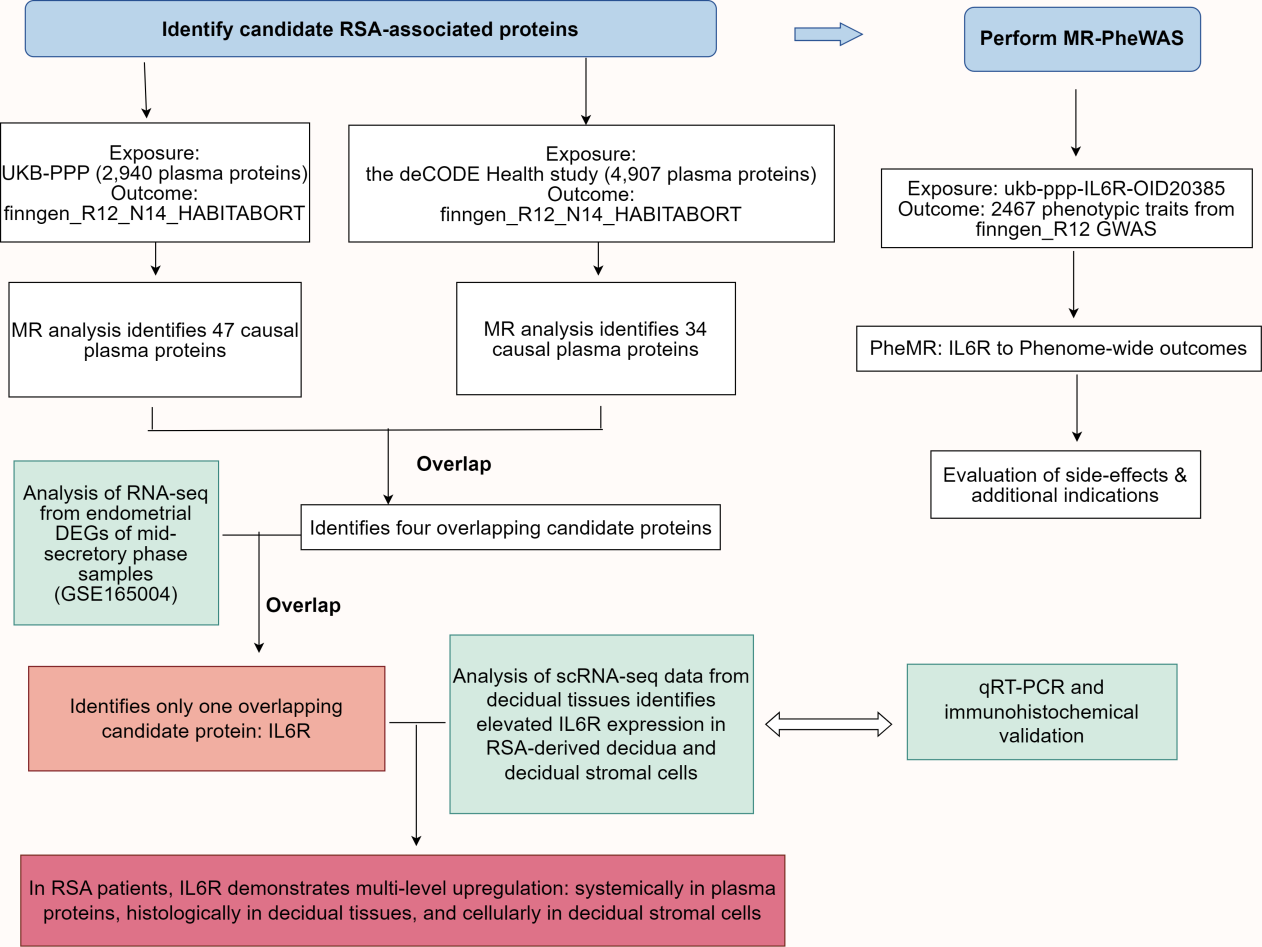
Schematic representation of the research design. MR analysis of plasma proteomics data from the UK Biobank Protein Plasma Project (UKB-PPP) and the deCODE Health Study identified 47 and 34 causal plasma proteins associated with RSA risk, respectively. The genetic data for RSA were obtained from the FinnGen R12_N14_HABITABORT dataset. Four candidate proteins were identified by overlapping the two datasets, with IL6R being the only candidate protein that overlapped with DEGs from mid-secretory phase endometrial RNA-seq data of RSA patients and controls (GSE165004). Reanalysis of single-cell RNA-seq data from the decidual tissues identified elevated IL6R expression in RSA-derived decidua and stromal cells. The IL6R expression was significantly upregulated at both the plasma protein and decidua mRNA levels in RSA patients. Finally, MR-PheWAS was performed using the ukb-ppp-IL6R-OID20385 dataset as the exposure variable and 2467 phenotypic traits from the finngen_R12 GWAS dataset as the outcome to evaluate the potential side effects and additional indications for IL6R as a drug target for RSA. DEGs, Differentially expressed genes; SNP, Single nucleotide polymorphism; IL6R, interleukin 6 receptor; UKB-PPP, UK Biobank Pharma Proteomics Project; RSA, Recurrent spontaneous abortion; RNA-seq, RNA sequencing; MR, Mendelian randomization; MR-PheWAS, MR-Phenome-wide association study; GWAS, genome-wide association study.

Comprehensive genome-wide association study (GWAS) summary data were derived from the original investigations wherein all participants had provided informed consent. We adhered to the STROBE-MR guidelines to ensure transparent and comprehensive reporting of our MR study (17), which included providing details on the study design, data sources, and the assumptions underlying the MR analysis. Initially, we employed two independent two-sample MR analyses to assess the causal effects of plasma proteins from the UK Biobank and Icelandic cohorts on RSA. Subsequently, proteins displaying concordant causal effects (*P* < 0.05 in both cohorts) were intersected with differentially expressed genes (DEGs) identified from RSA case-control RNA sequencing (RNA-seq) data using Ensembl gene ID matching. Consequently, the interleukin 6 receptor (IL6R) was identified as a unique candidate protein. Single-cell RNA-seq (scRNA-seq) analysis further examined the expression of IL6R in both the RSA group and the control group. Finally, a phenome-wide association study (PheWAS) was conducted to comprehensively evaluate the role of IL6R across diverse phenotypes, to evaluate the safety implications and therapeutic potential of targeting IL6R for RSA treatment, as well as to explore its potential as a therapeutic target. **Figure 1** depicts a flowchart that systematically outlines the research process employed in this study.

### Data sources for RSA

Summary statistics for RSA from a GWAS were acquired from FinnGen_R12 (https://r12.risteys.finngen.fi/). The dataset (GWAS ID: finngen_R12_N14_HABITABORT), released in 2024, investigates habitual abortion, commonly known as RSA. This study encompassed 130,971 individuals of Finnish descent, comprising 811 cases and 130,160 control participants, with 20088611 SNPs meeting the quality control parameters.

### Data sources for plasma pQTLs

We identified index cis-SNPs associated with the plasma protein levels at a genome-wide significance threshold (*P* < 5 × 10^-8^) to serve as IVs, utilizing data from two extensive GWASs: the deCODE Health study (18) and the UK Biobank Pharma Proteomics Project (UKB-PPP) (19). The UKB-PPP performed proteomic profiling on blood plasma samples from 54,219 participants using the Olink platform (19). Cis-SNPs were characterized as single nucleotide polymorphisms (SNPs) located within 1 megabase of the gene that encodes the corresponding protein, with linkage disequilibrium (LD) assessed employing the European reference panel provided by the 1000 Genomes. For 2,940 proteins, a comprehensive mapping of pQTLs was performed, with the genomic location of pQTLs frequently being near the gene associated with the specific protein. In practical terms, a pQTL located close to its associated gene is called a “cis-pQTL,” assuming that it affects the gene through direct interaction. In the two-sample MR analysis, index cis-SNPs corresponding to 1,728 plasma proteins were derived from the deCODE Health study. This study involved the measurement of 4,907 aptamers in a cohort of 35,559 Icelandic individuals by using the SomaScan platform (18). Similarly, we selected index cis-SNPs as IVs for 1980 proteins from the UKB-PPP (19). Our findings highlight the overlapping proteins identified as genetic instruments from two separate analyses so as to evaluate the consistency of results obtained from different proteomic profiling platforms.

### Selection of IVs

In MR studies, IVs must adhere to the following three fundamental assumptions: (i) the relevance assumption, which posits that the IV exhibits a robust association with the exposure, typically indicated by an F-value of >10 to denote a strong correlation; (ii) the exclusivity assumption, which states that the IV should not influence the outcome; (iii) the independence assumption, which asserts that the IV must remain unassociated with any other confounding factors (20).

The instrumental variable selection protocol implemented the following three sequential filters (1): cis-pQTLs were operationally defined as genetic variants positioned within a proximal 2 Mb genomic window (spanning ±1 Mb from transcriptional initiation sites) (2); genome-wide significance threshold (*P* < 5 ×10^-8^) was applied to ensure high-confidence protein-SNP associations (3); LS pruning (r² < 0.001) was conducted to eliminate correlated variants, thereby preserving genetic instrument independence (4). The clumping window size for MR analyses of phenome-wide analysis was set to 10,000 kb. In contrast, a clumping window size of 1,000 kb was employed for proteome-wide MR (5); F-value calculated as (β2/se2) >10 to exclude weak IV bias (6); SNPs with a minor allele frequency (MAF) of 0.01 or less were not included (21, 22).

### MR analysis

The inverse-variance weighted (IVW) method is recognized as the most reliable technique for evaluating all IVs (23). Consequently, this approach was adopted as the primary method for analysis in the present study. Statistically significant outcomes were defined as those with *P* < 0.05. However, it is important to note that the conclusions drawn from the IVW method may be biased in the presence of a horizontal pleiotropy (23). To address this potential limitation, the analysis was further supported by employing MR–Egger regression and the weighted median method, both of which aimed to bolster the reliability of the findings. MR-Egger regression is known to produce trustworthy estimates, although it may be affected by outliers (24). To investigate the presence of a broader horizontal pleiotropy, we utilized the MR-PRESSO approach (25). The possibility of a directional pleiotropy was assessed by applying the intercept test from MR-Egger regression and a significant non-zero intercept (*P* < 0.05), indicating a systematic directional pleiotropy (26). Moreover, we conducted a leave-one-out sensitivity analysis to determine whether the IVW estimate was influenced by a single SNP (27). Cochran’s Q test was applied to validate the presence of multiple variants, with heterogeneity acknowledged at a significance level of *P* < 0.05 (28). All analyses were conducted by using the TwoSampleMR package (version 0.6.8), the MR package (version 0.8.0), and the MR Pleiotropy Residual Sum and Outlier (MR-PRESSO) package (version 1.0) within the R software environment (version 4.4.2; https://www.R-project.org).

### RNA-seq data analyses

The endometrial transcriptomic profiles analyzed in this investigation were derived from the publicly accessible Gene Expression Omnibus (GEO) repository with the accession number GSE165004 (12). This dataset, generated using the Illumina HiSeq 4000 system (platform identifier GPL16699), originally comprised endometrial tissue specimens from three distinct cohorts: 24 RSA cases, 24 individuals diagnosed with idiopathic infertility, and 24 reproductively competent females serving as reference subjects. Our analytical cohort was selectively restricted to 24 cases that met the diagnostic criteria for RSA and 24 demographically matched healthy controls with proven fertility. Endometrial samples were obtained during days 19–21 of the menstrual cycle. DEGs were screened using the GEO2R. Then, Gene Ontology (GO) and Kyoto Encyclopedia of Genes, Genomes (KEGG) analyses were conducted using the R statistical software (version 4.2.1) with the clusterProfiler package (v4.4.4) and ggplot2 package (3.4.4) (29). For the visualization of GO and KEGG results in bar charts, a more stringent selection criterion was applied, specifically |log2 fold change| > 0.585 and *P*adj < 0.05. Conversely, the chord diagram was generated using DEGs selected with the broader criterion of *P*adj < 0.05 only. The chord diagram not only quantitatively displayed changes in the gene expression but also highlighted the intensity of interactions among gene groups through the use of color and connection strength.

### scRNA-seq data reanalysis

The scRNA-seq data of first-trimester decidual tissues (6 RSA cases and 5 normal controls) from our previous investigation (30) were subjected to a secondary analysis to map the IL6R expression landscapes. The expression patterns of IL6R in decidua tissues and decidual stromal cells (DSCs), the main cell type in decidual tissues, between normal and RSA groups were visualized through violin plots generated by geom_violin and ggboxplot function performed using ggpubr v0.6.0. By employing the netVisual_chord method from CellChat, we could effectively visualize the communication patterns of Interleukin 6 signaling among various cell types. This approach can help illustrate how Interleukin 6 regulates signaling across senders, receivers, mediators, and influencers, thereby revealing its key role and the complexity of interactions within the cellular network.

### MR-PheWAS

PheWAS, commonly referred to as reverse GWAS, is a methodological approach used to examine associations between SNPs or phenotypes and a wide range of traits across the entire phenome (31). This method is particularly useful for assessing any potential side effects associated with the drug targets (31). The research utilized plasma proteins displaying positive MR results (IL6R, ukb-ppp-IL6R-OID20385), indicating exposure. The Finnish database (version R12), which includes 2467 phenotypic traits, provided the outcome data. To address the false-discovery rate in multiple comparisons, significance was set at an FDR-adjusted P < 0.05. SNPs associated with outcomes were excluded if their P value was below the Bonferroni-corrected threshold (P < 0.05/N. SNPs) in the MR-PheWAS analysis.

### Sample collection and ethics statement

This study was conducted in accordance with the Declaration of Helsinki and approved by the Medical Ethics Committee of The Third Affiliated Hospital of Guangzhou Medical University (Approval No. 202401). Written informed consent was obtained from all participants prior to tissue collection. The RSA cohort comprised women with two or more consecutive unexplained pregnancy losses following confirmed normal (both parental and fetal) karyotypes, excluding individuals with uterine anomalies, endocrine/metabolic disorders, autoimmune conditions, or infections. Control subjects were gestational age-matched women undergoing voluntary termination of confirmed viable intrauterine pregnancies without history of pregnancy losses. Demographic characteristics of the participants providing decidual specimens for molecular analyses, are comprehensively detailed in **Supplementary Table S1**. All GWAS datasets, RNA-seq and scRNA-seq data included in this analysis underwent ethical review and were approved by their respective institutional ethics boards, with written informed consent obtained from all participants.

### Quantitative real-time polymerase chain reaction

RNA extraction and qRT-PCR were conducted using RNAiso Plus and PrimeScript RT Reagent Kit for total RNA extraction and reverse transcription (TaKaRa, Japan). Gene expression was measured using TBGreen Premix Ex Taq II on a QuantStudio 3 Real-Time PCR System (Applied Biosystems, USA). Primer sequences are in **Supplementary Table S2**, and mRNA expression was normalized to the *RNA18SN1* housekeeping gene.

### Immunohistochemistry

Paraffin-embedded endometrial specimens from controls undergoing elective termination and RSA patients, underwent standardized immunohistochemical processing as described previously (32). Following formalin fixation and paraffin embedding, 5 *μ*m sections were mounted on charged slides. Antigen retrieval was performed using citrate buffer (pH 6.0) before incubation with rabbit polyclonal anti-IL6R antibody (Proteintech; 1:200 dilution) overnight at 4 °C. Detection employed secondary antibodies (Cell Signaling Technology; 1:500) with 60-minute room temperature incubation, counterstained with DAPI nuclear marker (Sigma-Aldrich; 1 *μ*g/mL).

### Statistical analysis

Image processing and figure composition were conducted using Adobe Photoshop (Version 7.0). Quantitative data from qRT-PCR and immunohistochemistry were analyzed through IBM SPSS (Version 21.0) and GraphPad Prism (Version 8.0) platforms, employing independent two-sample *t*-tests and nonparametric tests for group comparisons. Continuous variables are presented as mean ± standard deviation, with statistical significance defined as *P* < 0.05 (two-tailed probability threshold).

## Results

### Proteome-wide MR intersected with RNA-seq data identifies one plasma protein, IL6R, as a putative therapeutic target for RSA

**Figure 2:**
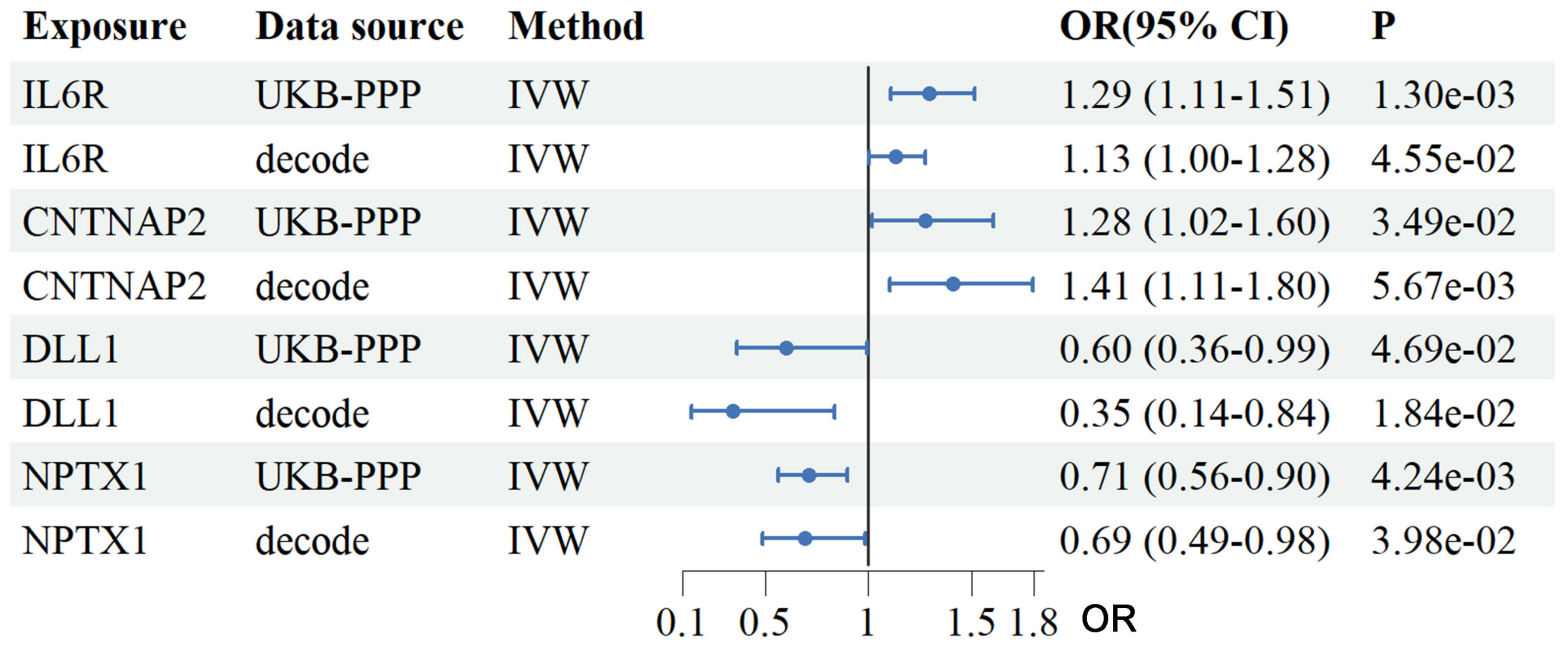
Analysis of the association between plasma proteins and the risk of RSA using the IVW method. The forest plot depicts the causal effects of plasma proteins on RSA risk based on a two-sample MR analysis using data from UKB-PPP and the Icelandic cohort. The four overlapping proteins identified were IL6R, CNTNAP2, DLL1 and NPTX1. OR and 95% CI were estimated using the IVW method. UKB-PPP, UK Biobank Prospective Proteomic Study; deCODE, Icelandic cohort; IVW, Inverse variance weighted; OR, Odds ratios; CI, confidence intervals.

Using two-sample MR we analyzed plasma proteomic data from two large cohorts: the Icelandic deCODE study (4,907 proteins; n = 35,559) and the UK Biobank-PPP (2,940 proteins; n = 54,219). In total, 34 proteins from the deCODE dataset and 47 from the UKB-PPP dataset were significantly associated with RSA risk (IVW *P* < 0.05, **Supplementary Tables S3**-**S6**). Intersection analysis identified four plasma proteins consistently associated with RSA across both cohorts: IL6R (UKB-PPP OR = 1.29, 95% CI: 1.11–1.51; deCODE OR = 1.13, 95% CI: 1.00–1.28), contactin associated protein 2 (CNTNAP2) (UKB-PPP OR = 1.28, 95% CI: 1.018–1.60; deCODE OR = 1.41, 95% CI: 1.11–1.80), Delta-like canonical Notch ligand 1(DLL1) (UKB-PPP OR = 0.60, 95% CI: 0.36–0.99; deCODE OR = 0.35, 95% CI: 0.14–0.84), and neuronal pentraxin-1 (NPTX1) (UKB-PPP OR = 0.71, 95% CI: 0.56–0.90; deCODE OR = 0.69, 95% CI: 0.49–0.98) (**Figure 2**).

**Figure 3:**
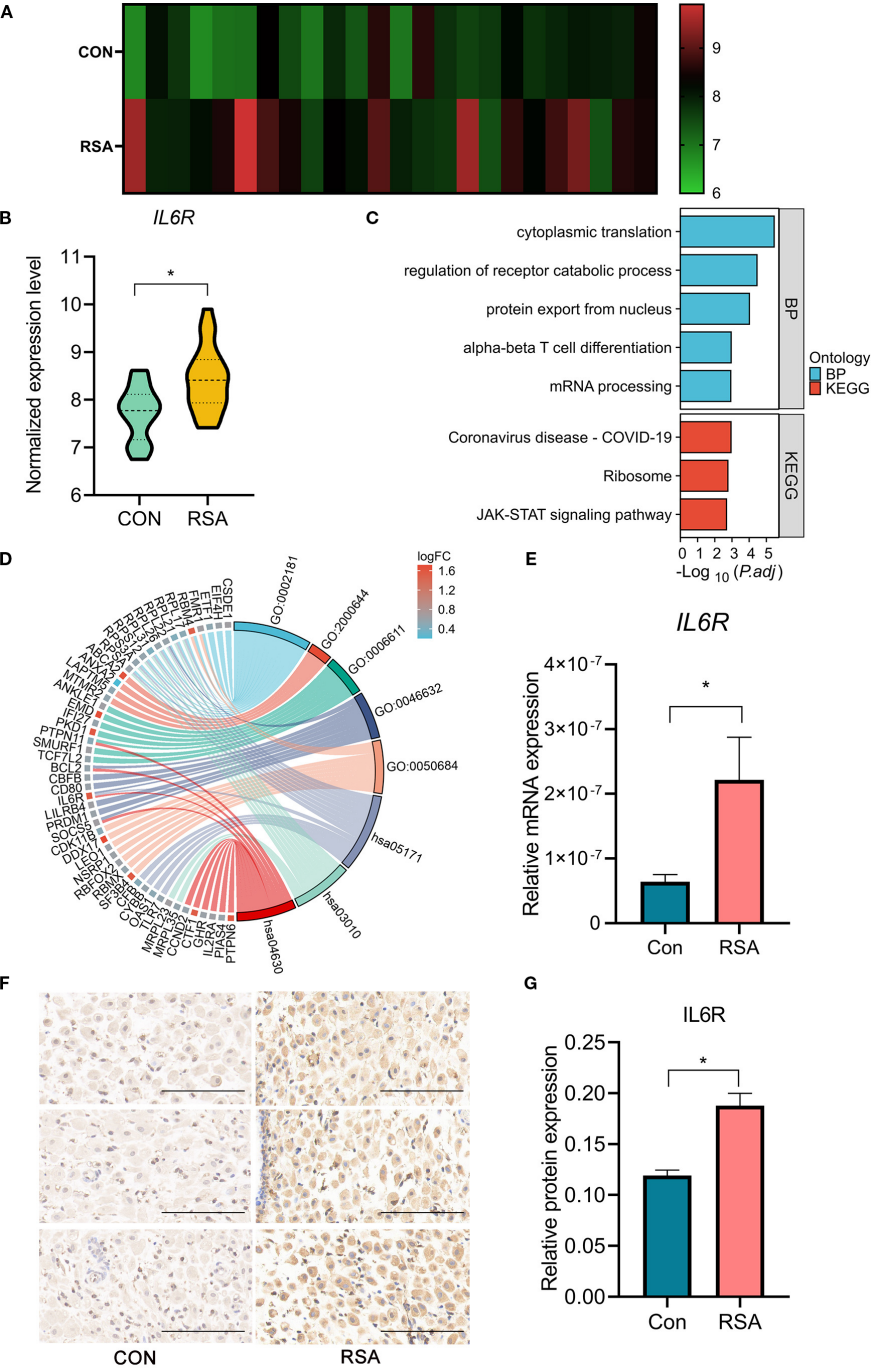
Comparative analysis of IL6R expression and pathway interactions in endometrial and decidual tissues of control group and RSA group. **(A)** Heatmap of IL6R expression in the mid-secretory endometria from RSA cases (n=24) compared to controls (n=24). Each row represents a sample, and color intensity indicates expression levels, with scale provided. **(B)** RNA-seq analysis of *IL6R* expression in mid-secretory endometrium derived from RSA group (n=24) compared to control group (n=24). *P < 0.05. **(C)** Bar chart displaying GO and KEGG pathway enrichment of DEGs in mid-secretory endometria derived from RSA cases compared to controls. **(D)** Chord Diagram of pathway-gene associations visualizes associations between DEGs and enriched biological processes and pathways. Ribbon widths indicate the strength of gene-pathway interactions, with significant links between IL6R, JAK-STAT signaling, and α-β T cell differentiation. **(E)** *IL6R* mRNA levels in deciduas from women with RSA and those with normal early pregnancies were analyzed using quantitative real-time polymerase chain reaction. The results were normalized to RNA18SN1 expression and are presented as the mean ± SD.*P < 0.05. n=18. **(F, G)** immunohistochemistry (IHC) staining and analysis of IL6R expression in early pregnancies deciduas from control group and RSA group. Scale bars,100*μ*m. n=13. IL6R, interleukin 6 receptor; CON, Control; RSA, recurrent spontaneous abortion. GO,0002181, cytoplasmic translation; GO,2000644, regulation of receptor catabolic process; GO,0006611, protein export from nucleus; GO,0046632, alpha-beta T cell differentiation; GO,0050684, regulation of mRNA processing; hsa05171, Coronavirus disease- COVID-19; hsa03010, Ribosome; hsa04630, JAK-STAT signaling pathway.

To further refine our identification of tissue-specific therapeutic targets, we integrated mid-secretory-phase endometrial RNA-seq data (GSE165004), identifying 359 differentially expressed genes (DEGs) (**Supplementary Table S7**), Among these, IL6R was the only candidate that overlapped with the four MR-identified plasma proteins associated with RSA risk. It demonstrated bidirectional consistency: elevated plasma levels were associated with increased disease risk, and there was concomitant upregulation in RSA endometrial tissues (**Figures 3A, B**). Consequently, IL6R was established as the prioritized therapeutic candidate.

Further transcriptomic analysis of endometrial tissues from RSA cases and controls identified a subset of DEGs enriched in biological pathways critical for immune regulation and cellular signaling. GO and KEGG analyses underscored significant enrichment in pathways such as α-β T cell differentiation and JAK-STAT signaling (**Figure 3C**). Notably, IL6R emerged as a pivotal gene within the α-β T cell differentiation pathway, suggesting its crucial role in modulating adaptive immune responses. A chord diagram further reinforced these associations, emphasizing the quantitative and functional interplay between IL6R and JAK-STAT signaling pathway (**Figure 3D**).

Furthermore, *IL6R* mRNA level was significantly upregulated in RSA decidua at the mRNA level (**Figure 3E**). In addition, immunohistochemistry further confirmed the significant increase in IL6R protein level in RSA decidua (**Figures 3F, G**).

**Figure 4:**
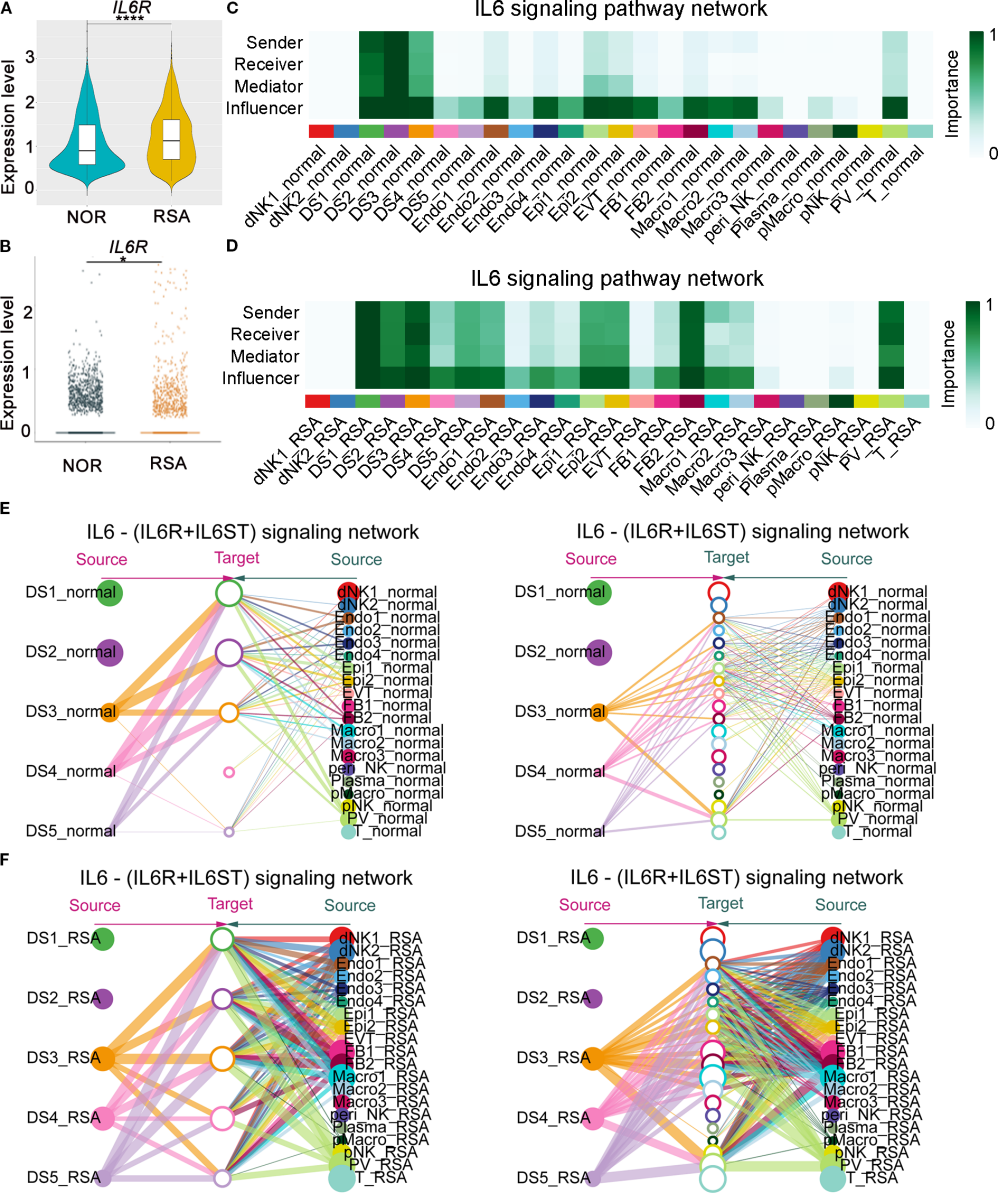
Abnormal upregulation of decidual IL6R mRNA in RSA patients was associated with IL6-signaling pathway dysregulation and compromised intercellular communication at the maternal-fetal interface. **(A)** Comparative single-cell RNA-seq (scRNA-seq) profiling of the IL6R transcript levels in decidual tissues from RSA patients (n = 6) versus gestational age-matched healthy controls (n = 5). The IL6R mRNA expression demonstrated a significant elevation in the RSA cohort (****P < 0.0001). **(B)** DSCs-specific analysis of scRNA-seq data demonstrated significantly elevated *IL6R* mRNA levels within DSCs populations from RSA patients (n = 6) when compared to gestational age-matched healthy controls (n = 5). **(C)** Analysis of scRNA-seq data revealed how different cell subtypes serve as senders, receivers, mediators, or influencers in the IL6-signaling pathway in the control group. The importance score (bar height) represents the communication probability (range 0–1), quantifying the relative strength of IL-6 ligand–receptor interactions between "sender" and "receiver" cell clusters. Higher values indicate stronger inferred communication. **(D)** Analysis of scRNA-seq data revealed how different cell subtypes serve as senders, receivers, mediators, or influencers in the IL6-signaling pathway in the RSA group. **(E)** Analysis of scRNA-seq data indicated the crosstalk between DSCs and the other subtypes in the control group, with a focus on the IL6-signaling pathway. Solid circles on the left represent different DS cell subpopulations, hollow circles in the center represent target cell subpopulations, and solid circles on the right represent other distinct cell subpopulations. The size of each circle is proportional to the number of cells within the corresponding subpopulation. Lines connecting nodes represent inferred significant ligand–receptor interactions, indicating potential cellular communication. The width of each line denotes the relative strength of the interaction. **(F)** Analysis of scRNA-seq data indicated the crosstalk between DSCs and other cellular subtypes in the RSA group, focusing on the IL6-signaling pathway. DS1-DS5, Decidual stromal cells; dNK1-dNK2, Decidual natural killer cells; Endo1-Endo4, Endometrial cells; Epi1-Epi2, Epithelial cells; FB1-FB2, Fibroblast cells; Macro1-Macro3, Macrophages; peri_NK, Peripheral blood natural killer cells; Plasma, Plasma cells; pMacro, placental macrophages; pNK, Placental natural killer cells; TPV, Trophoblast blood vessels; T, T cells.

Leveraging our published decidual scRNA-seq atlas, comprising first-trimester samples from five controls and six patients with RSA (30), we observed significantly elevated *IL6R* mRNA expression in the RSA cohort (**Figures 4A, B**). Furthermore, the communication model of IL6R among different cellular subtypes serving as senders, receivers, mediators, and influencers was disrupted, providing valuable insights into the molecular mechanisms underlying RSA pathogenesis (**Figures 4C, D**). Specifically, IL6 pathway crosstalk between DSCs and other cell populations was markedly enhanced in RSA, suggesting aberrant immune communication contributes to disease pathogenesis (**Figures 4E, F**).

Collectively, these findings demonstrate that *IL6R* is upregulated at both systemic and local tissue levels in RSA, marked by increased plasma protein levels and elevated expression in decidual and stromal cells.

### Phenome-wide evaluation of IL6R targeting

**Figure 5:**
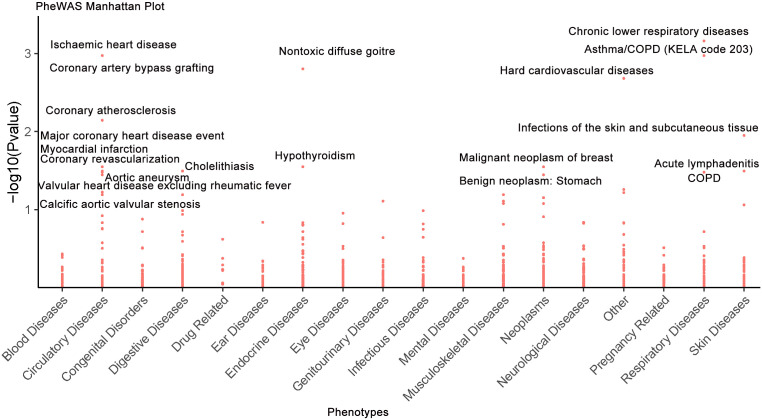
Phenome-wide causal estimates for IL6R perturbation are displayed, as derived from MR analyses of Finnish database clinical traits. The -log10(P-values) are plotted along the ordinate, with 20 labels applied to associations surpassing predefined statistical significance criteria (FDR < 0.05).

A comprehensive two-sample MR-PheWAS was performed using data from the FinnGen cohort, encompassing 784 clinical diagnoses, 17 disease categories, and 2,469 quantitative phenotypes (https://r12.finngen.fi/) (**Supplementary Table S8**). This analysis evaluated the effect of genetically predicted plasma IL6R concentrations on disease risk, with effect estimates representing changes in disease odds per standard deviation increase in IL6R levels. Associations were classified as detrimental if consistent with increased RSA risk, or protective if opposed. Sensitivity analyses using alternative MR methods confirmed the robustness of the observed associations (**Supplementary Table S9**). Detailed MR estimates with corresponding confidence intervals for all examined phenotypes are presented in the supplementary materials (**Supplementary Table S9**). At the Bonferroni-corrected significance threshold (*P* ≤ 6.38 × 10^−5^, α = 0.05/784), 20 significant associations were identified (**Figure 5**; **Supplementary Table S10**), including 8 potentially protective relationships (40%; **Supplementary Table S10**). Notably, IL6R exhibited pleiotropic protective effects against several conditions (*P* ≤ 6.38 × 10^−5^, α = 0.05/784), including benign neoplasm of the stomach, alcoholic gastritis, and multiple cardiovascular diseases (e.g., ischemic heart disease, coronary artery bypass grafting, aortic aneurysm, calcific aortic valve stenosis, major coronary events, coronary atherosclerosis, myocardial infarction, and valvular heart disease excluding rheumatic fever). Protective associations were also observed for hypothyroidism. Conversely, elevated IL6R levels were associated with increased risk of several respiratory system disorders (*P* ≤ 6.38 × 10^−5^, α = 0.05/784), such as asthma, chronic obstructive pulmonary disease (COPD), chronic lower respiratory diseases, nontoxic diffuse goiter, malignant neoplasm of breast, cholelithiasis, infections of the skin and subcutaneous tissue, and acute lymphadenitis (**Supplementary Table S10**). These findings underscore the broad physiological impact of *IL6R* modulation, highlighting both the therapeutic potential and the possible adverse effects of IL6R-targeted strategies in the treatment of RSA.

## Discussion

This study utilized an integrative MR framework to identify causal therapeutic plasma protein targets in RSA, highlighting IL6R as a central pathogenic factor.

Our study identified four plasma proteins with causal associations with RSA risk: IL6R and CNTNAP2 as pathogenic contributors, and DLL1 and NPTX1 as protective factors.

Our multi-omics approach underlines the pivotal role of IL6R in RSA pathogenesis through elevated protein levels and transcriptomic upregulation in reproductive tissues. IL6R’s central position in immune modulation links α-β T cell differentiation (33), and JAK-STAT mediated signal transduction, which is essential for decidualization (34, 35), with implications for cytokine storm mechanisms similar to severe COVID-19 (36).

scRNA-seq analysis revealed IL6R signaling upregulation in decidual stromal and immune cells, which may suggest a potential disruption in immune tolerance that is critical for early pregnancy (37). Specifically, impaired crosstalk between DSCs and uterine natural killer (uNK) cells via IL6R may compromise key immunomodulatory functions critical for placental development, as uNK-derived IL6 family cytokines are known to regulate trophoblast invasion and spiral artery remodeling (38). Moreover, emerging evidence suggests that elevated IL6 levels in specific immune cell clusters (39), may hinder M2 macrophage polarization. This disruption may contribute to the etiopathogenesis of RSA by impairing fetal-maternal immune tolerance.

Mechanistically, IL6R mediates inflammatory responses via its heterodimeric structure, influencing both membrane-bound and soluble pathways (36). Mechanistically, IL6R mediates inflammatory responses primarily through two distinct pathways: classical cis-signaling and trans-signaling (40). In classical cis-signaling, membrane-bound IL6R (mIL6R) on the cell surface binds IL6 and recruits the glycoprotein 130 (gp130) co-receptor, activating intracellular signaling cascades such as JAK-STAT pathways in cells expressing mIL6R. In contrast, trans-signaling involves the soluble form of IL6R (sIL6R), which is generated by proteolytic shedding or alternative splicing. The sIL6R-IL6 complex can bind to gp130 on cells that lack mIL6R, thereby broadening the cellular responsiveness to IL6 and contributing to chronic inflammation. The clinical tractability of IL6R, demonstrated in autoimmune diseases through inhibitors like tocilizumab and sarilumab (41, 42), underscores its therapeutic potential in RSA-related inflammation.

While CNTNAP2 is well known for its role in neural development (43), our MR analyses also implicated CNTNAP2 in RSA susceptibility, supported by array comparative genomic hybridization analysis showing significant 7q34q36.3 duplications encompassing CNTNAP2 in RSA-linked pathogenic CNVs (44). The protective effects of DLL1 appear to be mediated through its involvement in endometrial remodeling via Notch2 signaling essential for decidualization (45). This decidualization process is critical for successful implantation and pregnancy maintenance (32), and increasing evidence implicates impaired decidualization in RSA pathogenesis (30, 32, 46–48). NPTX1 plays a role in uterine biology, potentially affecting uterine receptivity and stability (49). Furthermore, treatment of human endometrial stromal cells with synthetic progestins under hypoxic conditions significantly upregulated NPTX1 secretion (50), indicating its potential role in promoting decidualization.

Our MR-PheWAS analysis revealed IL6R’s significant causal associations with diverse phenotypes (*P* ≤ 6.38 × 10^−5^, α = 0.05/784), including reduced risks of COPD and asthma, which are consistent with prior studies (51). Protective trends were also observed for gastritis (*P* ≤ 6.38 × 10^−5^, α = 0.05/784), aligning with previously published research (42). This supports the need for careful consideration of IL6R’s pleiotropic effects in therapy development.

This study has several strengths. First, utilizing MR minimizes confounding and reverse causation through genetic instruments. Second, MR acts as a “natural randomized controlled trial”, providing robust evidence without the ethical concerns. Last, integration of large-scale GWAS and proteomic datasets enhances statistical power and reliability.

Several methodological limitations should be considered when interpreting our findings. First, results may lack generalizability due to European-biased cohorts. Second, Unmeasured gene-environment interactions could modify risk estimates (52).Third, residual horizontal pleiotropy remains possible despite sensitivity analyses. Additionally, although our study emphasizes the role of IL6R in RSA, mechanistic insights into its molecular mechanisms and downstream signaling pathways, particularly its mediation of crosstalk between uNK cells and DSCs, remain insufficiently characterized. Future studies involving IL6R inhibitor trial, functional assays and crosstalk between uNK cells and DSCs, are required to validate its mechanistic pathways in RSA. Additionally, the absence of clinical validation may limit the translation of our research findings into clinical practice, potentially impacting the perceived reliability of the therapeutic target. Future research should focus on conducting clinical trials to validate the efficacy and safety of interventions based on these findings. Last, exploring the molecular mechanisms in more diverse populations could enhance the generalizability of the results and provide deeper insights into the biological underpinnings. In summary, while our findings provide significant evidence pointing to IL6R as a key factor in RSA and propose it as a therapeutic target, the generalizability of these results requires cautious interpretation. Further studies involving diverse populations, comprehensive environmental data, functional assays, IL6R inhibitor-related experiment, and clinical validation are essential to confirm and extend the applicability of these findings to broader RSA patient groups and to develop effective, personalized treatment strategies.

## Conclusion

This study advances the understanding of RSA by integrating genetic, proteomic, and transcriptomic analyses to uncover the causal role of IL6R as a key pathogenic factor. This was consistently validated across various biological levels, showing upregulation in plasma, decidual tissues, and decidual stromal cells of RSA patients. Additionally, a MR-PheWAS approach was used to explore the broader implications of targeting IL6R, assessing potential side effects and other therapeutic applications. Our results highlight IL6R as a vital therapeutic target for RSA, suggesting new directions for translational research and clinical strategi.

## Data Availability

The original contributions presented in the study are included in the article/**Supplementary Material**. Further inquiries can be directed to the corresponding authors.
